# The role of intrinsic dynamics and noise for neural encoding and synchronization

**DOI:** 10.1186/1471-2202-12-S1-P109

**Published:** 2011-07-18

**Authors:** Christian Finke, Hans A Braun, Ulrike Feudel

**Affiliations:** 1 Institute for Chemistry and Biology of the Marine Environment, Carl von Ossietzky Univ. of Oldenburg, Germany; 2 Neurodynamics Group, Institute of Physiology, Philipps University of Marburg, Germany

## 

Transitions from tonic firing (single spikes) to burst discharges (impulse groups) play an important role for neuronal information processing (sensory encoding, information binding) and are closely associated with neuronal synchronization in many physiological processes (hormone release, sleep-wake cycles) as well as in disease (Parkinson, epilepsy). Synchronization is typically considered to depend on the network connectivity. The intrinsic dynamics of individual neurons are mostly neglected. However, in computer simulations we have seen that transitions from tonic firing to burst discharges can significantly facilitate synchronization without any changes of the coupling strength [1,2]. Thereby, it is not really surprising that periodically bursting neurons synchronize. The more interesting question is why the same neurons need much higher coupling strengths for synchronization when they are operating in a likewise periodic but tonic firing mode. Remarkably, the tonic firing mode also appeared to be much more sensitive to noise [3] which, in terms of phase space, can be related to deviations of the trajectories in the vicinity of a saddle point [4]. Our study shall help to understand under which conditions these particular features appear.

We have used a simplified Hodgkin-Huxley-type approach to implement different types of model neurons for the examination of their synchronization properties especially in the tonic firing mode. Starting point was a four-dimensional model neuron for spike-generation with subthreshold oscillations which can be tuned to a diversity of impulse pattern [5] and which exhibits the above described features. For comparison, we have examined HH-type model neurons without subthreshold oscillations but pacemaker properties.

When two identical, periodically firing neurons in the same dynamic state are connected via gap junctions (diffusive coupling) it should be expected that they are synchronizing at infinitely low coupling strengths in all periodically firing regimes. Indeed, this is the case in the examined model neurons – with only one exception as mentioned above. The subthreshold currents of the oscillatory subsystem, although not recognisable in the tonic firing regime, are introducing a dynamic instability on which the particular noise sensitivity is built up and which likewise prevents immediate synchronization. The input from neighboured neurons can be considered as a disturbance which rather induces random phase fluctuations than in-phase synchronisation.

The interdependencies between subthreshold and spike generating currents and their different time scales seem to be the necessary conditions for this behaviour but only the overlapping of their activation range, bringing the trajectories close to a saddle point, provides sufficient conditions.

## Conclusion

The model mimics quite well the experimentally observed impulse pattern and their relations to neuronal synchronisation, indicating that alterations of the neurons’ intrinsic dynamics can play an important role for synchronization also in real physiological systems. It is a particular challenge is to examine such phenomena in experimental studies. From the computational point of view, it needs to be analysed whether these noise and synchronisation effects are specific features of the here examined model [5] or general properties of model neurons which can be tuned from bursting to a pacemaker-like tonic firing mode. Such studies also can help to further elucidate from what kind of dynamics these specific features arise and, physiologically most important, under which conditions they can be expected.
